# Network-based exploration of 4-(phenylsulfonyl)morpholine molecules for metastatic triple-negative breast cancer suppression

**DOI:** 10.1371/journal.pcbi.1014132

**Published:** 2026-03-31

**Authors:** Jung-Chen Su, Chen-Ling Lee, Fan-Wei Yang, Yan-Chih Chen, Te-Lun Mai

**Affiliations:** 1 Department of Pharmacy, College of Pharmaceutical Sciences, National Yang Ming Chiao Tung University, Taipei, Taiwan; 2 Department of Life Science, College of Life Science, National Taiwan University, Taipei, Taiwan; 3 Center for Computational and Systems Biology, National Taiwan University, Taipei, Taiwan; Tecnológico de Monterrey: Tecnologico de Monterrey, MEXICO

## Abstract

Triple-negative breast cancer (TNBC) is an aggressive and heterogeneous subtype of breast cancer, with limited treatment options due to the absence of estrogen receptors, progesterone receptors, and human epidermal growth factor receptor 2 (HER2) expression. This characteristic renders TNBC resistant to hormone-based and HER2-targeted therapies, leaving cytotoxic chemotherapy as the predominant strategy and highlighting the urgency for novel interventions. In this study, we investigated the mechanism of action of GL24, a potent 4-(phenylsulfonyl)morpholine-based small molecule with selective tumor suppression effects on metastatic TNBC cells, while being ineffective against TNBC cells derived from the primary tumor site, using gene co-expression analysis. By considering the distinct phenotypic responses induced by GL24, we tailored our co-expression analysis approach, selecting gene pairs that exhibited differential co-expression in effective cells while excluding gene pairs that also showed differential patterns in non-effective cells. Constructing a co-expression network from these differential pairs, followed by enrichment analysis and functional annotation, revealed specific gene interactions and molecular pathways associated with GL24-mediated TNBC inhibition. These insights supported the previously established findings that showed convergence on apoptosis based on differentially expressed genes, while also providing complementary information by highlighting pathways involved in metabolic alterations, proliferation, and migration or invasion. This expanded understanding advances the knowledge of the mechanisms of GL24 in combating TNBC.

## Author summary

Triple-negative breast cancer (TNBC) is an aggressive form of the disease that often spreads and lacks three common drug targets. We previously found that GL24, a small molecule we designed, suppresses the growth of metastatic tumor cells more strongly than cells from the primary tumor site. To understand how GL24 works, we looked not only at individual genes but at how genes work together in pairs. We asked a simple question: which gene pairs become linked, or lose their link, only in the cells that respond to GL24, and not in the cells that do not? By building networks from these selective gene-gene pairs, we uncovered signals related to metabolic change, cell growth, and cell migration and invasion. These network patterns complement standard single-gene tests and offer testable ideas for how GL24 is effective against metastatic TNBC cells. More broadly, we share a simple, phenotype-filtered strategy: focus on gene relationships that track with the trait of interest (here, response to GL24) rather than all correlations. This approach is practical for small laboratory studies and may help guide future drug development.

## Introduction

Triple-negative breast cancer (TNBC) is an extremely aggressive subtype of breast cancer with the characteristics of complex biology and heterogeneity [1], a high tendency of tumor metastasis and recurrence, and poor long-term outcomes [2]. Unlike other breast cancer subtypes, TNBC lacks the expression of estrogen receptors, progesterone receptors, and human epidermal growth factor receptor 2 (HER2). This feature makes TNBC unresponsive to traditional hormone therapies or HER2-targeted treatments, posing a challenge in treatment selection. As a result, cytotoxic chemotherapy remains the primary treatment for both early- and advanced-stage TNBC [1]. Although there have been some advancements in the field of TNBC treatment, including the use of immunotherapy (immune checkpoint inhibitors such as PD-1 and PD-L1 inhibitors) and targeted therapies [Poly (ADP-ribose) polymerase inhibitors such as olaparib and talazoparib], achieving ideal therapeutic outcomes remains elusive. Therefore, discovering novel therapeutic agents and elucidating the molecular pathways involved in TNBC are crucial for overcoming the limitations of current treatments and improving patient outcomes.

The analysis of transcriptomic data generated from high-throughput RNA sequencing is widely employed in drug research to unveil mechanisms of action (MOA) and therapeutic targets, identify genes associated with drug sensitivity or resistance, and predict potential beneficial or adverse effects in pharmacogenomics [3,4]. The identification of differentially expressed genes (DEGs) followed by functional enrichment analysis represents a fundamental approach, which reveals pathway changes induced by drug compound treatments [4–9]. In our previous work [10], we developed a novel 4-(phenylsulfonyl)morpholine derivative, GL24, which specifically inhibited the growth of TNBC cell lines derived from metastatic lesions, with limited effects on those originating from primary sites. The DEGs from the transcriptomic data of the two effective cell lines (MDA-MB-231 and MDA-MB-157) and the one non-effective cell line (Hs 578T) were identified and analyzed to elucidate the MOA underlying the growth suppression effects triggered by GL24.

However, DEG analysis relies exclusively on genes that exhibit significant changes under treatment, which presents some limitations. For example, modest changes in the expression of all genes within a pathway may exert a greater influence on activation or deactivation than a significant change in a single gene, leading to an underestimation of pathway effects in DEG analysis [11]. To overcome these challenges, various methodologies have been developed [12–14]. Among them, the module inference method uses clustering algorithms to identify modules of co-expressed genes within the gene co-expression network, emphasizing correlation or dependency relationships over significant expression changes [14]. Such network-based analyses complement DEG analysis by offering additional insights into gene relationships and the related annotated functions.

**Fig 1:**
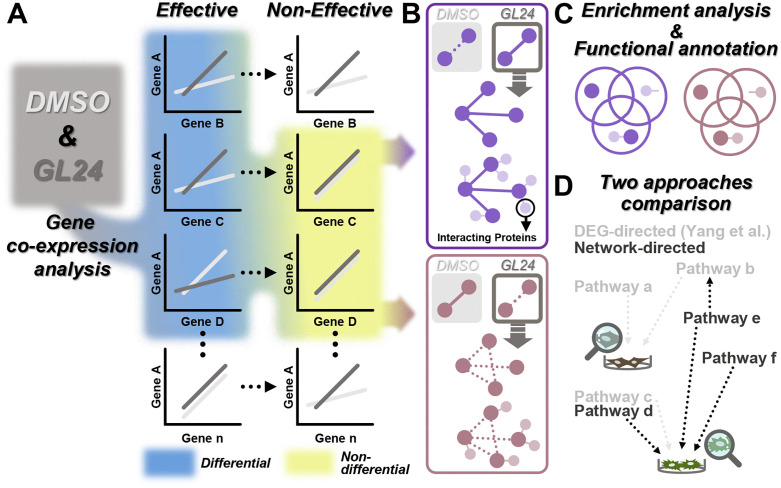
The schematic workflow for elucidating the molecular actions of GL24 based on gene co-expression analysis. **(A)** Transcriptomic data obtained from DMSO and GL24 treatments in TNBC cells were subjected to gene co-expression analysis. Differential co-expression gene pairs (blue) were identified in effective cells, while these same gene pairs were required to be non-differential co-expression gene pairs (yellow) in non-effective cells. **(B)** The differential co-expression gene pairs meeting the criteria (blue and yellow in panel A) were classified into two categories following GL24 treatment: the formation (top, purple) or disappearance (bottom, brown) of gene associations. Network analysis identified clusters corresponding to these changes. Large dark circles (purple or brown) represented cluster genes. Solid purple lines indicated the formation of gene associations, while dashed brown lines represented the disappearance of gene associations. Then, these cluster genes and their interacting proteins (small circles in light purple or brown), either together or separately, underwent enrichment analysis and functional annotation **(C)**. **(D)** The results of the gene co-expression analysis in this study were compared with those derived from DEG-directed analysis (Yang et al.) to clarify the differences in pathway identification between these two transcriptome-related approaches for elucidating the mechanisms of GL24. Cells labeled with different colors represented distinct phenotypic traits.

To achieve a more comprehensive and precise understanding of the MOA of GL24, we performed gene co-expression analysis using transcriptomic data from the above two GL24-effective cell lines and one non-effective cell line under solvent control and GL24 treatment. Differential co-expression gene pairs were identified based on their differential correlation patterns in GL24-effective cell lines, while being non-differential in non-effective cells (Fig 1A). A gene co-expression network was constructed using only these differential pairs or including their potential interacting proteins (Fig 1B), followed by pathway enrichment analysis and functional annotation (Fig 1C). This approach uncovered specific gene associations and pathways that may mediate the anti-tumor effects of GL24, providing robust support and valuable complementation to the previously developed DEG-directed method [10] (Fig 1D).

## Methods

### Gene expression data processing

The gene expression data processing steps followed the methodology outlined in our previous study [10]. The raw expression data were obtained from our previously generated dataset, which consisted of 30 samples divided into six groups, each containing five samples [10]. Briefly, gene read counts were quantified using the RSEM tool (version 1.2.28) [15], with alignment performed by the STAR aligner (version 2.7.10a) [16] under default parameters. The analyses were based on the human reference genome (GRCh38) and GENCODE annotation (release 41). As a sensitivity check, we compared the results obtained using GRCh38 with GENCODE release 38 to those generated using Telomere-to-Telomere human genome (T2T-CHM13v2.0) with EBI GENCODEv38 r2 to evaluate the potential impact of reference genome choice on mapping performance [17], and observed only minor differences in mapping metrics (0.25 ± 0.04%). Gene expression levels were normalized and measured as transcripts per kilobase million (TPM) [18] based on the mapping results from GRCh38.

### Co-expression matrix construction and edge definition

To construct the co-expression matrix, the Pearson correlation coefficient was employed to measure the expression relationship between any two genes based on their R values, *P* values, and empirical *P* values. Given the limited sample size (*n* = 5 per treatment group), the R value itself was not used as a selection criterion. The sign of *r* was stored as additional information for interpretation, but was not used in the clustering step.

To assess whether observed correlations exceeded those expected by random chance, empirical *P* values were calculated for each gene pair using a permutation-based approach. In each treatment group, labels in the gene expression matrix were randomly shuffled 200 times to generate a null distribution of correlation-based *P* values. For each gene pair with an observed uncorrected $P_{g}$, the empirical *P* value was defined as the proportion of permuted *P* values ($P_{i}$) smaller than $P_{g}$, calculated as $(\sum_{i=\text{1}}^{\text{200}}number\;of\;\left(P_{i}<\;P_{g}\right))/\text{200}$, where $P_{i}$ refers to the *P* value obtained from the i-th permutation. This permutation-based procedure generated an empirical null distribution, enabling us to determine whether the observed correlations were stronger than those expected by random chance without relying entirely on parametric assumptions.

To define edges within the co-expression network based on these statistical evaluations, we applied the following criteria. A significant (correlated) co-expression relationship between two genes was defined only when both the *P* value and the permutation-based empirical *P* value (200 permutations) were < 0.005. Gene pairs with *P* > 0.05 were considered non-significant (uncorrelated), and those in which co-expression could not be calculated because one or both genes showed no expression across all five replicates were labeled as “NA (not available).” Gene pairs that did not fall into any of these three categories were excluded from further analysis to avoid ambiguous results, reduce the likelihood of false positives, and enhance the separation between defined categories. In all cases, the classification (significant, non-significant, or NA) was made only when at least three distinct (x, y) expression pairs were available for the gene pair. To directly link gene-pair relationships to the anti-TNBC phenotype induced by GL24, we further restricted our analysis to gene pairs that showed differential scenarios in both GL24-effective cell lines but remained non-differential in the non-effective cell line. Under this framework, edges in a network represent: (1) gene pairs whose relationships shifted from non-significant or NA under DMSO to significant after GL24 treatment; (2) the opposite transition, from significant under DMSO to non-significant or NA after treatment; or (3) gene pairs that remained significant before and after treatment but exhibited a significant change in correlation strength between the two treatments. In the network visualization, the first two types of edges are shown as solid and dashed lines, respectively, and only these two types were observed in our results.

### Network modeling and analysis

We modeled the co-expression networks as undirected and unweighted graphs, defining each edge as 1 if any of the three differential scenarios was satisfied and 0 otherwise. Given the limited sample size, we avoided using |*r*| as an edge weight by design, aiming to minimize noise that could arise from small-sample variability.

To mitigate potential network sparsity resulting from our scenarios, we defined “clusters” simply as connected components, that is, groups of genes connected to each other through edges. Connected components were identified using the igraph::clusters function [19], and their size distributions were summarized to characterize the overall network architecture. This component-based strategy allowed us to capture biologically meaningful patterns without relying on clustering algorithms that might impose artificial partitions on a sparsely connected network.

Finally, nodes with a degree ≥ 10 were defined as hubs. These hub genes represent highly connected nodes within the network and may serve as key regulators in GL24-induced transcriptional responses.

### Functional enrichment analysis

Functional enrichment analysis was performed using the R package clusterProfiler (version 4.12.6), focusing on Gene Ontology (GO) terms and Kyoto Encyclopedia of Genes and Genomes (KEGG) pathways [20]. For GO analysis, only the “Biological Process” (BP) ontology was used in this study. To extend the analysis by including interacting proteins associated with the identified gene pairs, the STRING database [21] was utilized to access the information on known and predicted protein-protein interactions. Interacting proteins were identified for each cluster based on an interaction score > 950 (confidence > 0.95), ensuring high reliability of the interactions.

## Results

### Identification of differential co-expression gene pairs related to GL24-induced anti-TNBC properties through gene co-expression analysis

Gene co-expression analysis is influenced by two key parameters: the co-expression metric and the threshold for significance. Correlation coefficients (R) are commonly used to represent co-expression, with thresholds based on R values, *P* values, or both. In our analysis, we observed that when *P* values were below 0.1, the corresponding absolute R values were remarkably high (R ≥ 0.8), and these R values were expected to be even higher under the commonly accepted significance threshold of *P* < 0.05. This finding reflected the limitations of relying solely on R-value-based thresholds, which may be due to a relatively small sample size or low data variability. Therefore, we used *P* values as the sole criterion, defining gene pairs with *P* values < 0.005 and empirical *P* values < 0.005 as significant (correlated gene pairs), while gene pairs with *P* values > 0.05 were classified as non-significant (uncorrelated gene pairs). In addition, we assigned “not available” (NA) to gene pairs without measurable expression. NA cases were retained, as a shift from NA under DMSO to significant under GL24 indicated that a previously unevaluable relationship became detectable, thereby classifying the pair as a “differential co-expression gene pair.” This parameter design enabled the identification of reliable co-expression relationships, providing the foundation for downstream analyses of GL24-induced changes in gene networks (see Methods for details).

**Fig 2:**
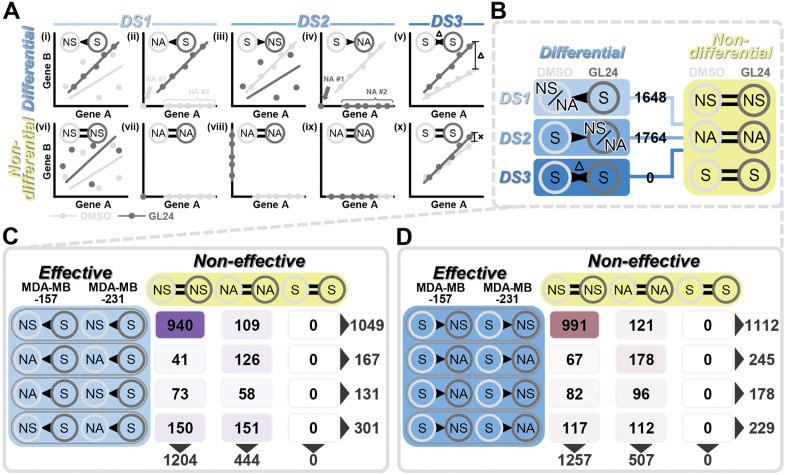
Schematic representation and analysis of differential and non-differential co-expression gene pairs in effective and non-effective TNBC cells. **(A)** A schematic representation of potential scenarios illustrating differential (i to v) and non-differential (vi to x) co-expression gene pairs, using Gene A and Gene B as examples. Differential scenarios are shown as follows: **(i-ii)** DS1, differential scenario 1 (light blue); **(iii-iv)** DS2, differential scenario 2 (medium blue); and **(v)** DS3, differential scenario 3 (dark blue). Light and dark grey represented DMSO and GL24 treatments, respectively. Dots denoted replicates, while lines depicted regression lines. NS referred to non-significant, S to significant, and NA to not available. NA #1 and NA #2 referred to two potential cases where data were not available. Hollow triangles in panel A (v) indicated differential R values, while cross marks in panel A (x) indicated no differential R values. **(B)** The number of gene pairs showing differential co-expression in effective cells and non-differential co-expression in non-effective cells was calculated for each differential scenario (DS1 to DS3). Blue represented the scenarios involving differential co-expression (from light blue to dark blue, corresponding to DS1 to DS3), while yellow represented the scenarios involving non-differential co-expression. For example, without distinguishing between specific non-differential co-expression patterns in non-effective cells, 1,648 gene pairs met the DS1 criteria in effective cells, where DMSO treatment was either non-significant (NS) or not available (NA), and GL24 treatment was significant **(S)**. **(C-D)** Based on panel B, the number of gene pairs involved in the possible differential combinations of two effective cell lines and the three non-differential co-expression patterns in non-effective cells was listed. A total of 12 potential conditions were considered. **(C)** Gene pairs involved in DS1 (purple). **(D)** Gene pairs involved in DS2 (brown).

For these differential co-expression gene pairs, three possible scenarios were proposed. The first scenario involved a shift from not correlated to correlated (hereafter referred to as “DS1”, from the initial letters of “differential scenario 1”). For example, in the previously mentioned case, DMSO treatment resulted in a non-significant or NA correlation, while GL24 treatment led to a significant correlation [Fig 2A, (i) and (ii) as schematic examples]. The second scenario represented the opposite shift (hereafter “DS2”), from correlated to not correlated, where a significant correlation became non-significant or NA [Fig 2A, (iii) and (iv) as schematic examples]. The third scenario involved a significant correlation under both DMSO and GL24 treatments, but with a difference in R values (hereafter “DS3”) [Fig 2A, (v) as a schematic example]. To elucidate the gene functions associated with each scenario, changes in co-expression relationships under DMSO and GL24 treatments were required to align with the same scenario in both effective TNBC cell lines (MDA-MB-157 and MDA-MB-231). However, due to the high heterogeneity of TNBC, identical patterns between the two effective cell lines were not mandated within a given scenario. As a result, four possible combinations were proposed within the DS1: (1) both MDA-MB-157 and MDA-MB-231 showed a shift from non-significant to significant, (2) both cell lines shifted from NA to significant, (3) MDA-MB-157 shifted from NA to significant while MDA-MB-231 shifted from non-significant to significant, or (4) MDA-MB-157 shifted from non-significant to significant while MDA-MB-231 shifted from NA to significant. Similar to the DS1, four possible combinations across the two effective cell lines could also occur in the DS2.

If these differential co-expression gene pairs were observed in both effective and non-effective cell lines, the changes in gene pair correlations would not be specifically attributable to the anti-cancer phenotype induced by GL24. Thus, when a differential co-expression gene pair was identified in two effective cell lines, it needed to simultaneously be classified as a “non-differential co-expression gene pair” in a non-effective cell line. Three types of non-differential co-expression gene pair patterns could be expected. The first two patterns, both categorized as not correlated, involved either non-significant correlations under both DMSO and GL24 treatments [Fig 2A, (vi) as a schematic example] or were designated as NA in both treatments [Fig 2A, (vii) to (ix) as schematic examples]. The third pattern showed significant correlations under both treatments, with no differential change in R values [Fig 2A, (x) as a schematic example].

Among the total 3,412 gene pairs that met all of the criteria above, 1,648 pairs (2,264 genes) fell into the DS1, 1,764 pairs (2,333 genes) into the DS2, and none into the DS3 (Fig 2B and S1 Table). In the gene pairs belonging to DS1, the predominant pattern across all combinations was that the two effective cell lines exhibited the same transition from non-significant to significant, while these gene pairs were simultaneously designated as non-significant in the non-effective cell lines for both DMSO and GL24 treatments, comprising 940 pairs and accounting for 57.0% of all DS1 gene pairs (Fig 2C). A similar pattern was observed in the gene pairs belonging to DS2, where the most common combination, seen in 991 gene pairs (56.2%), involved two effective cell lines showing an identical shift from significant to non-significant, with these gene pairs also classified as non-significant in the non-effective cell lines under both DMSO and GL24 treatments (Fig 2D). In differential gene pairs, the most common combinations involved two effective cell lines transitioning between non-significant and significant states in both DS1 and DS2, representing 63.6% (1,049 out of 1,648) in DS1 and 63.0% (1,112 out of 1,764) in DS2 (Fig 2C and 2D). In non-differential gene pairs, the outcome most frequently observed was non-significant results for both treatments, accounting for 73% (1,204 out of 1,648) in DS1 and 71.2% (1,257 out of 1,764) in DS2 (Fig 2C and 2D). NA cases were also prevalent, comprising 43.0% (708 out of 1,648) of the gene pairs in DS1 and 43.8% (773 out of 1,764) in DS2, regardless of whether they were classified as differential or non-differential gene pairs, or both (Fig 2C and 2D). No non-differential gene pairs, exhibiting significant correlations under DMSO and GL24 treatments and showing no differential change in R values, were detected in either DS1 or DS2 (Fig 2C and 2D).

### Network analysis of gene pairs involved in DS1 and DS2

**Fig 3:**
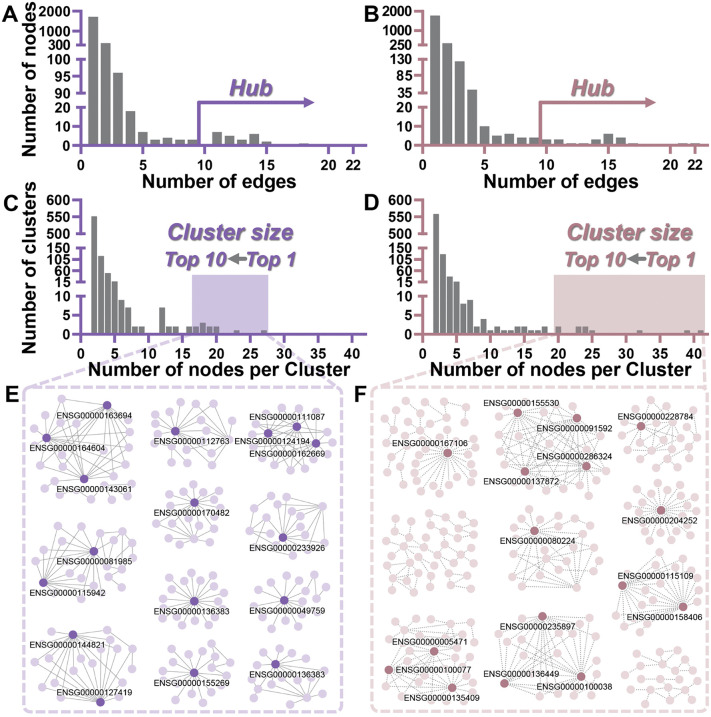
Node degree distribution, cluster size analysis, and network visualization of co-expression networks modeled as undirected and unweighted graphs for DS1 and DS2. **(A-B)** The bar plots depicted the distribution of node degree (number of edges) in DS1 **(A)** and DS2 **(B)**. The x-axis indicated the number of edges per node, while the y-axis showed the corresponding node count. Nodes with a high degree (≥10) were referred to as hubs. **(C-D)** The bar plots showed the distribution of cluster sizes in DS1 **(C)** and DS2 **(D)**. The x-axis represented the number of nodes per cluster, while the y-axis indicated the number of clusters of each size. The shaded region indicated the top 10 clusters with the largest number of nodes. **(E-F)** The network topologies for the top 10 clusters in DS1 and DS2 were shown in **(E)** and **(F)**, respectively. The nodes within each cluster were represented as light purple in DS1 and light brown in DS2. Hub genes were highlighted using dark purple for DS1 and dark brown for DS2. The solid and dashed lines represented the formation and disappearance of gene relationships in panels **(E)** and **(F)**, respectively.

To better understand the gene interactions involved in DS1 and DS2, network analysis was conducted, focusing on gene connectivity and cluster sizes. First, we analyzed the degree distribution, defining nodes with a degree ≥ 10 as hubs. This analysis identified 24 hubs in each of DS1 and DS2, representing 1.1% of the total nodes in DS1 and 1.0% in DS2 (Fig 3A and 3B). One hub was shared between both scenarios, resulting in a total of 47 unique genes. Next, we examined the size distribution of clusters, including the number of nodes in the top 10 clusters and the percentage of clusters containing only two to three genes. The top 10 clusters in DS1 (11 clusters, due to the same cluster size) and DS2 (10 clusters) comprised 216 and 271 nodes, representing 9.5% and 11.6% of all nodes, respectively (Fig 3C-3F and S1 Data). The largest cluster in DS1 contained 27 nodes, while in DS2 it contained 41 nodes (Fig 3C and 3D). Most clusters were small, with 85.8% in DS1 and 87.5% in DS2 consisting of only 2–3 nodes (Fig 3C and 3D and S2 Table).

Notably, gene pairs in DS1 transitioned from a lack of correlation to significant correlation following GL24 treatment, as network analysis identified newly established gene clusters. In contrast, gene pairs in DS2 shifted from significant correlation to no correlation, reflecting the disruption of gene associations and the dissolution of clusters induced by GL24 treatment.

### Functional enrichment analysis of clusters in DS1 and DS2

To understand the biological processes involved with the DS1 and DS2 cluster genes, we performed functional enrichment analysis of the top 10 clusters identified in each group, applying Gene Ontology (GO) and Kyoto Encyclopedia of Genes and Genomes (KEGG) (S3 Table). The GO analysis of the DS1 cluster genes revealed the enrichment of two GO terms related to membrane repolarization (GO:0086009) and membrane depolarization (GO:0051899), which describe the processes of membrane potential regulation, both involving ion channel activity and cellular electrical signaling. Cancer cells are characterized by membrane depolarization as a distinct bioelectrical property compared to their normal counterparts [22,23], which may indicate the activation of voltage-gated calcium channels and the subsequent influx of calcium ions, ultimately influencing cellular behaviors such as proliferation, apoptosis, and migration [23–26]. Membrane repolarization regulates the opposite process of depolarization and typically involves the efflux of potassium ions or the closure of calcium and sodium channels, returning the membrane potential to a more negative and stable level [27]. Both inhibiting and hyperactivating the potassium ion channel protein (Kv11.1) have been shown to lead to distinct cellular outcomes in cancer cells, with inhibition activating apoptosis and hyperactivation triggering a senescent program [28]. On the other hand, KEGG analysis revealed that DS2 cluster genes were enriched in starch and sucrose metabolism (hsa00500) and carbohydrate digestion and absorption (hsa04973). Both pathways involve the breakdown of carbohydrates into monosaccharides (e.g., glucose) and the subsequent absorption, linking them to carbohydrate metabolism. Cancer cells often rely on increased glycolysis for rapid energy production and the generation of intermediates essential for tumor growth, known as the Warburg effect [29,30]. Targeting carbohydrate metabolism has emerged as a potential therapeutic strategy in cancer treatment [31–34].

**Fig 4:**
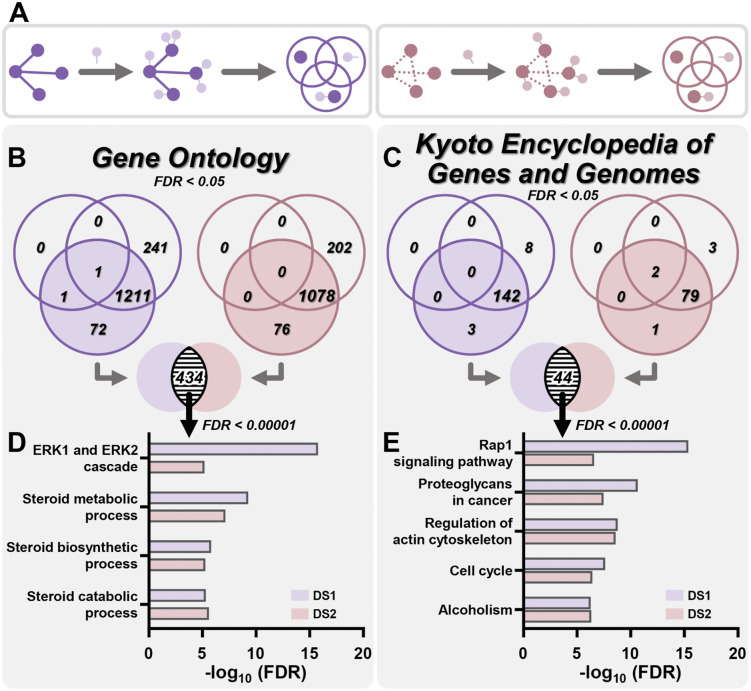
Enrichment analysis of GO terms and KEGG pathways for the top 10 cluster genes and their interacting partners in DS1 and DS2. **(A)** The STRING database was used to identify cluster-interacting proteins for the top 10 clusters. On the left, DS1; on the right, DS2. GO and KEGG analyses were performed on the cluster genes (dark purple, DS1; dark brown, DS2; top left in the schematic Venn diagrams), their interacting proteins (light purple, DS1; light brown, DS2; top right in the schematic Venn diagrams), or the combination of cluster genes and their interacting partners (bottom in the schematic Venn diagrams). **(B-C)** The Venn diagrams illustrated the enrichment counts of GO terms **(B)** and KEGG pathways **(C)** across the three aforementioned groups in DS1 (purple) and DS2 (brown) (FDR < 0.05). Intersection counts represented the overlap between DS1 and DS2 in cluster genes and their interacting partners. **(D-E)** The most significant GO terms **(D)** and KEGG pathways **(E)** in these intersections were displayed, with -log_10_(FDR) values for DS1 (purple) and DS2 (brown). An FDR < 0.00001 indicated high significance.

Based on the assumption that similar expression profiles suggest similar functions [14], proteins interacting with the top 10 clusters were incorporated into the functional enrichment analysis to increase the likelihood of identifying pathways with shared functions. Cluster-interacting proteins were identified using the STRING database to retrieve known and predicted protein-protein interactions. In addition to the aforementioned analysis of cluster genes, we included two additional groups: (1) using only cluster-interacting proteins and (2) combining cluster genes with their interacting proteins (Fig 4A). In both GO and KEGG analyses, some pathways were enriched exclusively by cluster-interacting proteins, while some were solely enriched by the combination of cluster genes and their interacting proteins (Fig 4B and 4C). For example, in DS1, 241 GO terms were enriched specifically in the cluster-interacting proteins group, while 72 GO terms were enriched uniquely in the cluster genes with their interacting proteins group (Fig 4B, *left*). Given the importance of cluster gene functions, we then focused on the group combining cluster genes and their interacting proteins. Pathway terms enriched in both DS1 and DS2 suggested that, regardless of whether gene pair correlations were formed or lost due to GL24, these genes participated in the same pathways, highlighting the importance of these functional pathways. In total, 434 GO terms and 44 KEGG pathways were identified (Fig 4B and 4C). We further raised the significance threshold from FDR < 0.05 to FDR < 0.00001 to identify representative pathways in the combination of cluster genes and their interacting protein groups (S4 Table). Under these conditions, four GO terms were enriched: ERK1 and ERK2 cascade (GO:0070371), steroid metabolic process (GO:0008202), steroid biosynthetic process (GO:0006694), and steroid catabolic process (GO:0006706) (Fig 4D). In addition, five KEGG pathways were also enriched: Rap1 signaling pathway (hsa04015), proteoglycans in cancer (hsa05205), regulation of actin cytoskeleton (hsa04810), cell cycle (hsa04110), and alcoholism (hsa05034) (Fig 4E). Among these GO and KEGG enriched terms, the ERK1 and ERK2 cascade and the Rap1 signaling pathway were most significant in either DS1 or DS2 (Fig 4D and 4E). Extracellular signal-regulated kinase 1/2 (ERK1/2), part of the mitogen-activated protein kinase (MAPK) family, mediates cellular processes such as cell growth, migration, and differentiation [35]. Abnormal activation of ERK1/2 frequently occurs in cancer, highlighting its significance as a therapeutic target [36]. Inhibitors targeting upstream regulators of ERK1/2, such as MEK (mitogen-activated protein kinase kinase) and BRAF (a serine/threonine-protein kinase), have been developed to counteract the oncogenic effects of ERK1/2 [37–40]. Under certain conditions, ERK1/2 promotes apoptosis, reflecting a double-edged sword nature in cancer treatment strategies [41]. Ras-associated protein-1 (Rap1) is a small GTPase belonging to the Ras-related protein family, which is activated or deactivated by binding to guanosine triphosphate (GTP) or guanosine diphosphate (GDP), respectively [42]. Two isoforms of Rap1 (Rap1A and Rap1B) have been implicated in various cancers, including roles in cell migration, invasion, and the progression of aggressive cancer phenotypes [42,43]. In colorectal cancer, for example, high expression of Rap1A has been linked to tumor cell proliferation via the PTEN/FOXO3/CCND1 pathway [44]. In addition, regulators of Rap1 activity, such as Rap1GAP, are frequently impaired during tumor progression. Rap1GAP normally functions to negatively regulate Rap1, and its loss results in elevated levels of active Rap1-GTP [42]. In breast ductal carcinoma in situ, the downregulation of Rap1GAP has been shown to drive the transformation into invasive ductal carcinoma [42,45]. These findings highlight the significance of Rap1 and its regulators in cancer-related processes, making it a potential target for therapeutic strategies.

## Discussion

### The enriched pathways comparison between the two approaches

**Fig 5:**
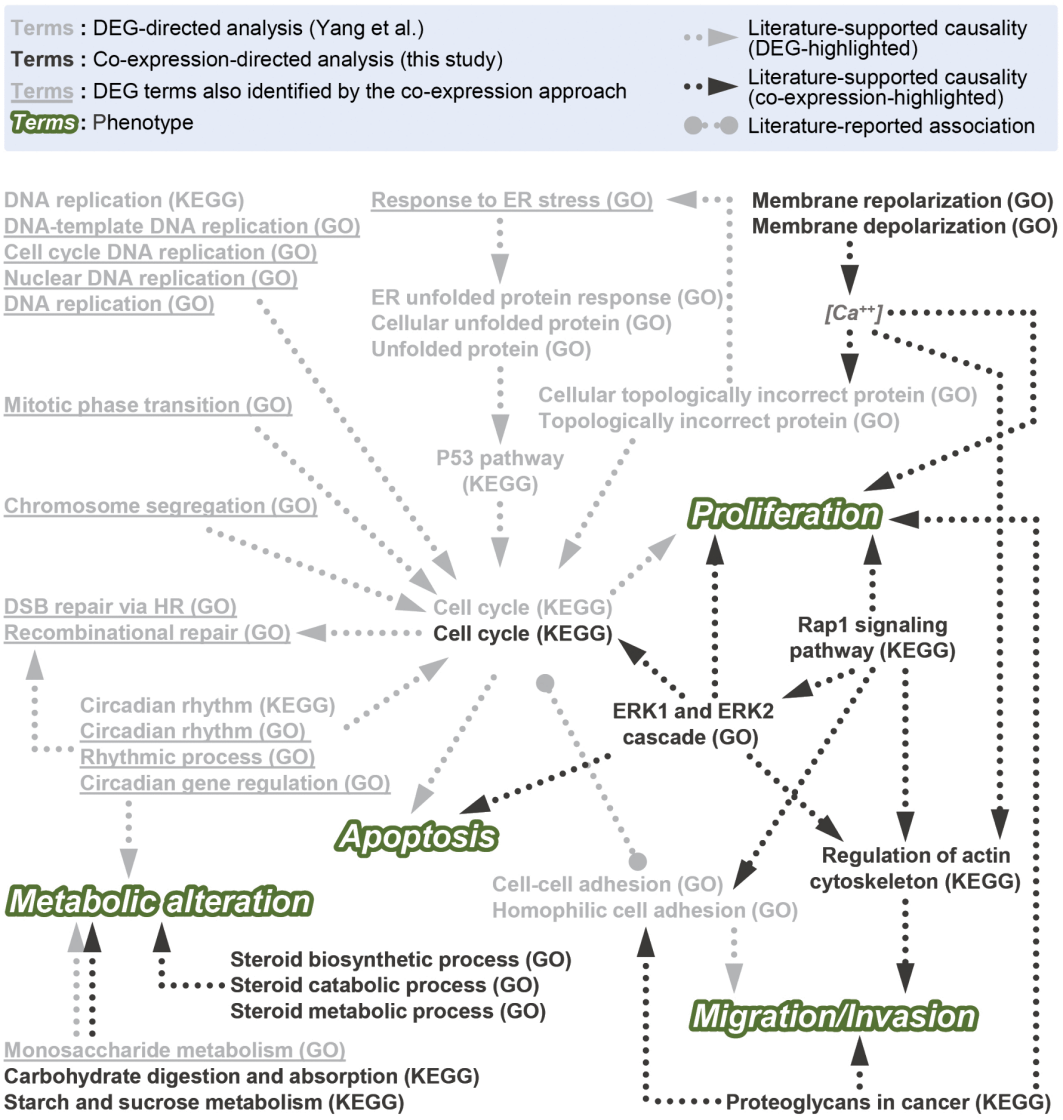
A comparison of the enriched pathways triggered by GL24 based on DEG-directed (Yang et al.) and co-expression-directed (this study) analyses. The light grey GO and KEGG terms represented the results from the DEG-directed analysis. The dark grey GO and KEGG terms represented the primary results obtained from the co-expression-directed approach. These included: (1) analyses of genes from the top 10 clusters in either DS1 or DS2, and (2) analyses of genes from the top 10 clusters combined with their potentially associated proteins. For case (2), only the intersection between DS1 and DS2 was shown, with an FDR < 0.00001. The light grey terms with underscores indicated the DEG-directed GO terms that could be identified using the co-expression-directed approach in either DS1 or DS2 (FDR < 0.05). The dashed arrows represented the causality supported by studies in the literature. The dotted line connecting solid circles represented a possible interaction between the two terms. The white text highlighted with a green background represented phenotypic traits potentially associated with these terms.

The comparison of the functional enrichment analysis results from the previous study, which employed DEG-directed analysis [10], and this study, which utilized co-expression-directed analysis, revealed notable differences (Fig 5). The top 10 up- and down-regulated GO terms and four significant KEGG pathways identified through DEG analysis were selected to represent the results of DEG-directed functional enrichment analysis (Fig 5, light grey text). Most of these GO and KEGG terms led to or were regulated by the cell cycle (Fig 5, light grey text with light grey dashed arrows). As the cell cycle is well-known for its role in apoptosis regulation [46,47], we validated the credibility of the DEG-directed analysis in the previous study by examining apoptosis activity following GL24 treatment [10].

One notable difference was that, unlike the DEG-directed analysis, where multiple enriched terms converged on a single pathway or biological process, the significant enrichment terms from the co-expression-directed analysis revealed a more intricate interplay among pathways (Fig 5, dark grey text with dark grey dashed arrows). The crosstalk between migration/invasion and proliferation was taken as an example. Actin served as a central mediator in cell migration by driving the formation of protrusions such as lamellipodia and filopodia. This process relied on actin polymerization, regulated by actin-binding proteins that coordinated cytoskeletal reorganization to support cell movement [48]. Rap1 signaling activated several Rho family members to regulate actin cytoskeleton dynamics, as well as integrin- and cadherin-mediated cell-cell adhesion, both of which influenced migration in distinct ways [42,43]. Additionally, Rap1 participated in the activation or inhibition of ERK activity to control cell proliferation [42,43,49]. ERK was found to drive actin polymerization and adhesion turnover for cell movement [50]. Proteoglycans consisted of a core protein covalently linked to glycosaminoglycan chains and were part of the extracellular matrix and the cell glycocalyx [51,52]. They were critical for maintaining structural integrity, regulating cell signaling, and mediating cell adhesion to control cell migration and proliferation [51–55]. For example, the intracellular proteoglycan serglycin facilitated the migration of non-small cell lung cancer cells through interactions between its glycosaminoglycans and CD44 [55]. Heparan sulfate proteoglycans interacted with growth factors through their heparan sulfate chains to regulate cell growth [53,54]. The regulation of membrane potential also exhibited crosstalk between migration/invasion and proliferation (Fig 5). Calcium ion concentration was regulated by membrane repolarization and depolarization processes, while aberrant or dysregulated calcium ion signaling has been associated with increased cell proliferation [25] and the modulation of actin-binding proteins involved in cell migration [26].

The second observed difference was that the co-expression-directed analysis either provided additional support for existing term categories identified by DEG-directed analysis or introduced new term categories. For instance, in the DEG-directed analysis, metabolic alterations were influenced by circadian rhythm-related pathways (circadian rhythm, rhythmic process, and circadian gene regulation; we consolidated the terms with similar functions into a unified term category) and monosaccharide metabolism (Fig 5 and ref [10]). The co-expression-directed analysis provided additional support for monosaccharide metabolism by identifying pathways such as starch and sucrose metabolism and carbohydrate digestion and absorption, all involved in glucose metabolism, thereby strengthening the existing association between metabolic alterations and glucose metabolism (Fig 5). Furthermore, based on the DEG-directed analysis, we inferred that GL24 induced incorrect proteins in TNBC cells, leading to ER stress and the accumulation of unfolded proteins (Fig 5 and ref [10]). The co-expression analysis in this study identified membrane repolarization and depolarization as key factors associated with changes in calcium concentration, which may contribute to protein misfolding and subsequently trigger ER stress [56]. These findings suggested potential upstream signaling pathways that could support the DEG-directed results. Another example of this difference was observed in the cell cycle-apoptosis interaction. While the DEG-directed analysis primarily highlighted the cell cycle, the co-expression-directed analysis also identified this pathway. Moreover, ERK was found to regulate the cell cycle [35] and, under specific conditions, promote apoptosis [41]. Although the co-expression-directed analysis did not emphasize the cell cycle-apoptosis relationship as a primary focus, its findings provided additional support, reinforcing the observations from the DEG-directed analysis.

In addition to the cell cycle being identified as significant by both approaches, 13 out of 20 DEG-directed GO terms were found among the enriched terms in the co-expression-directed DS1 or DS2 analyses (Fig 5, light grey text with underlines). These terms were, however, less prominently ranked in the co-expression-directed analysis, indicating differences in the significantly enriched pathways prioritized by the two approaches. Furthermore, seven significant DEG-directed GO terms—ER unfolded protein response, cellular unfolded protein, unfolded protein, cellular topologically incorrect protein, topologically incorrect protein, cell-cell adhesion, and homophilic cell adhesion—were not identified in the co-expression-directed analysis. This observation underscored the distinct characteristics of each approach. By integrating these two approaches, a more comprehensive understanding of the MOA of a drug or compound could be achieved.

Both DEG-directed and co-expression-directed enrichment analyses are correlation-based and cannot establish causation, so the findings should be regarded as hypothesis-generating and will require further validation through experiments or appropriate drug-perturbation datasets [57]. While the precise molecular target of GL24 and the mechanisms underlying its selective activity in metastatic TNBC cells remain undefined, several of the enriched pathways identified in our analysis have previously been reported to sustain proliferation and migration in cancer cells [25,42,43,48–51,53–55]. This overlap raises the possibility that GL24 suppresses TNBC growth by perturbing these pathways, and that one or more pathway molecules, or their direct or indirect interactors, may represent its molecular target. Our differential co-expression analysis also suggests potential directions for identifying critical regulators from two perspectives: network topology and pathway context. From a topology-based viewpoint, highly connected DS1/DS2 hub genes occupy central positions and co-vary with many genes, making them plausible candidate regulators within the correlation network. Accordingly, the top three hubs in DS1 (ENSG00000163995, ENSG00000257335, and ENSG00000144821) and DS2 (ENSG00000204252, ENSG00000167106, and ENSG00000158406), together with a shared hub (ENSG00000205089) that exhibited hub properties in both scenarios (S2 Table), constitute a prioritized set. Notably, prior studies have linked these genes to cancer-relevant phenotypes [58–71] that are consistent with the anti-cancer axes inferred for GL24 in our analysis, including proliferation, apoptosis, migration/invasion, and metabolic alterations (Fig 5). From a pathway-guided perspective, incorporating interacting proteins extends the analysis beyond cluster-intrinsic functions to a broader signaling context, increasing the likelihood of capturing pathway-associated regulators and core components. Using stringent criteria shared by DS1 and DS2 (FDR < 0.00001), the ERK1 and ERK2 cascade (GO:0070371) and Rap1 signaling pathway (hsa04015) emerged as representative signals (Fig 4). Pathway-guided candidates may be considered by focusing on canonical core components that also appear in the top 10 clusters in DS1 and DS2. Under these criteria, MAPK1 (ENSG00000100030; ERK2) and the small GTPase RAP1A (ENSG00000116473) represent plausible candidates for the ERK1 and ERK2 cascade and Rap1 signaling pathway, respectively. We further speculate that TNBC cells derived from metastatic lesions, relative to those from primary tumors, may exhibit differences in the expression or functionality of these critical regulators, thereby rendering them more vulnerable to GL24 treatment. Future studies using targeted perturbation approaches, such as loss- and gain-of-function experiments, combined with phenotypic assays assessing proliferation [10], apoptosis [10], migration/invasion [72], and metabolic alterations [31], will be required to determine whether these candidates causally mediate GL24 sensitivity and action. Despite these uncertainties, the pronounced efficacy of GL24 in metastatic TNBC cells highlights its therapeutic potential, together with that of other 4-(phenylsulfonyl)morpholine derivatives, for targeting metastatic disease.

### Data-driven strategy for identifying differential co-expression gene pairs in response to GL24-induced anti-TNBC properties

The sensitivity of different TNBC cell lines to GL24 treatment varied significantly, with the half-maximal inhibitory concentration differing by more than 10-fold between effective and non-effective cell lines [10]. Leveraging this distinct GL24-induced phenotype, we incorporated it into the design of our co-expression analysis. Co-expression gene pairs were selected based on their differential co-expression in the two effective cell lines, while remaining non-differential in the non-effective cell line. We believed that only gene pairs meeting these criteria could truly represent the gene pairs relevant to GL24-mediated anti-TNBC effects, forming the core concept of our strategy (Fig 1). If the requirement for differential co-expression gene pairs to be non-differential in the non-effective cell line were removed, 4,589 pairs would be identified, representing a 34.5% increase compared to the 3,412 pairs identified under the current conditions (Fig 2B). This increase included 1,177 additional pairs unrelated to the GL24-suppressed TNBC phenotype. Notably, DEGs identified in the non-effective cell line constituted only ~0.1% of those in the two effective cell lines [10], and were previously deemed negligible in our DEG analysis [10]. This underscored the crucial role of the criteria in improving the accuracy of gene co-expression analysis and its subsequent interpretations.

This stringent design also addressed a methodological concern relevant to the later use of STRING. Because STRING integrates some cancer-related functional information, its application is often considered to risk circular reasoning, where enrichment for cancer pathways may be artificially reinforced by prior annotation. Without our phenotype filter, many gene pairs would appear differential across both effective and non-effective cell lines, and those gene pairs could not be specifically linked to the anti-cancer phenotype induced by GL24. When analyzed with STRING, such pairs could generate spurious enrichment for cancer pathways driven by STRING’s built-in annotations, thereby masking the fact that they are not phenotype-specific. By contrast, the applied filter ensured that the retained differential gene pairs were phenotype-specific, so STRING served primarily to broaden the network context rather than to redefine their cancer relevance. In addition, the functional themes identified after STRING expansion were supported by enrichment results obtained directly from data-driven gene sets without STRING expansion, including cluster genes alone and an independent DEG-based analysis, even though the specific GO/KEGG terms were not necessarily identical (Fig 5). This observation suggests that the main pathway signals were not solely driven by STRING annotations. Nevertheless, some residual risk of circular reasoning is inherent to STRING, and this limitation should be considered when interpreting our enrichment results.

### Methodological limitations of the small-n design

In this study, the small sample size (*n* = 5 per treatment group) represents an important limitation, as it reduces statistical power and increases the influence of random variation, which in turn may undermine reproducibility. Although five replicates are relatively larger than what is typically reported in exploratory RNA-seq studies [73,74], the design still falls within the small-n category. Under such conditions, standard genome-wide false discovery rate adjustment is not appropriate, since with only five replicates per treatment group, such correction would be overly stringent and likely remove many potentially meaningful signals [75,76]. To address this limitation, we applied multiple safeguards in our analysis design to mitigate the risks associated with the limited sample size and ensure that our conclusions remain robust and statistically credible. Specifically, we required both *P* values and permutation-based empirical *P* values from 200 label permutations to be < 0.005 to classify a pair as correlated. Pairs were classified as uncorrelated if the uncorrected *P* value was > 0.05, while those in intermediate ranges (e.g., uncorrected *P* < 0.005 but empirical *P* > 0.05) were excluded. By incorporating empirical *P* values and by strictly defining correlated and uncorrelated pairs while discarding intermediate pairs, we reduced potentially false positives. In addition, we required at least three valid data points per gene pair, and correlations that could not be calculated (NA) were retained as informative rather than discarded, to ensure reliable estimation. Finally, we imposed a phenotype filter, retaining only gene pairs that were differential in both GL24-effective cell lines while non-differential in the non-effective cell line, thereby strengthening the biological relevance of the findings.

One further consequence of this stringent filtering strategy, in combination with the limited sample size, is the absence of DS3 gene pairs (Fig 2B). DS3 represents gene pairs that remain significantly co-expressed under both DMSO and GL24 treatments but differ in correlation strength, whereas DS1 and DS2 reflect the gain or loss of significant co-expression between the two treatment groups. With only *n* = 5 per treatment group, the statistical power is limited for detecting correlations, and it becomes even weaker for detecting changes in correlation strength, making DS3 much less likely to be observed compared with DS1/DS2. In addition, DS3 requires four significant events under both DMSO and GL24 treatment across the two effective cell lines, while DS1/DS2 requires only two, making the probability of observing DS3 inherently lower. We therefore interpret the absence of DS3 as more likely a consequence of limited statistical power and strict analytical criteria, although the possibility of biological rigidity cannot be excluded. We anticipate that such patterns may become detectable with larger sample sizes.

### The comparison with existing co-expression methods

To evaluate our approach alongside existing methods, we compared it with several representative co-expression analysis workflows, including WGCNA [77], GeneCoEx [78], CoExp [79,80], and GeCoNet-Tool [81]. These tools typically follow a module-based design, in which networks are constructed across conditions (treatment groups) and subsequently analyzed for module-phenotype associations. In contrast, our method was designed to identify phenotype-specific changes at the gene-pair level, pinpointing cases where two genes become correlated or lose correlation specifically under the phenotype of interest. This framework integrates two statistical thresholds (*P* and permutation-based *P* values), strict data quality requirements, in which the classification (significant, non-significant, or NA) was made only when at least three valid expression pairs were available for a given gene pair, and phenotype-based filters to ensure that the detected changes are specific to the GL24-responsive context and absent in non-responsive conditions.

Applying all four workflows to the same RNA-seq dataset (Methods in S1 Text) revealed substantial differences in their outputs. As shown in S1A, S1C, and S1D Fig, WGCNA, CoExp, and GeCoNet-Tool identified a very large number of gene pairs in both DS1 and DS2 categories, involving 14,329, 14,329, and 10,461 genes, respectively, with each method covering more than 50% of the human protein-coding genome (S1 Fig). Such extensive coverage suggests that a considerable proportion of these associations may not specifically reflect the GL24-induced anti-TNBC phenotype. In contrast, GeneCoEx retained fewer than 100 genes (S1 Fig), which poses challenges for downstream enrichment analysis due to the limited size of the gene set.

These discrepancies can be partly explained by methodological differences. All four workflows rely on correlation as the fundamental measure, and with a small sample size (*n* = 5 per treatment group), correlation estimates are susceptible to random noise and require high |*r*| values to reach significance. When |*r*| cutoffs are not sufficiently stringent, correlation-based thresholds may bias results toward spurious associations, contributing to the large number of gene pairs identified by WGCNA, CoExp, and GeCoNet-Tool. This observation is consistent with the recommended use of WGCNA, which typically requires 15–20 samples for reliable network construction [82]. In contrast, GeneCoEx applies more stringent filters, such as Benjamini-Hochberg-adjusted *P* values and empirically defined |*r*| thresholds, to prioritize high-confidence signals. However, such criteria are optimized for datasets with many treatment conditions [78], and in our small-sample setting, they may have been overly restrictive, explaining the limited number of retained genes.

Although the results differ substantially from those obtained with our approach, we interpret this divergence as a reflection of distinct design philosophies rather than methodological superiority. Existing workflows were not originally developed for small-sample, phenotype-anchored gene-pair identification, which explains why their default behaviors may not align with our analytical objectives. With appropriate parameter tuning and application to their intended contexts, their performance would likely improve, and the number of detected genes could become more reasonable. Overall, these results highlight the importance of choosing analytical approaches that best fit the biological question and the limitations of the available data.

### Phenotype-filtered differential co-expression framework

Our analysis illustrates a strategy that can be generalized as a Phenotype-Filtered Differential Co-Expression Framework. This framework applies a phenotype filter that defines gene pairs as phenotype-specific only when they are differential in the phenotype of interest (Phenotype A) while remaining non-differential in a baseline or reference phenotype (Phenotype B). This safeguard reduces the likelihood of capturing broadly present correlations that are not specifically linked to the phenotype under investigation. The principle can be extended to other contexts, such as responder versus non-responder groups in clinical studies, or pathological versus physiological processes.

To operationalize this framework, it is necessary to specify how Phenotypes A and B are represented in the analysis. In this framework, each phenotype requires at least two treatment groups (for example, DMSO and GL24 in our study) to determine whether it represents a differential or non-differential scenario, with the two groups together forming what we refer to as a treatment pair. In practice, the number of treatment pairs assigned to each phenotype is flexible. The treatment pairs assigned to Phenotype A range from one to multiple, whereas those for Phenotype B range from zero to multiple. A greater total number of treatment pairs increases the robustness and confidence of the results. In our analysis, each cell line provided one treatment pair, with Phenotype A consisting of two pairs derived from two effective TNBC cell lines (MDA-MB-231 and MDA-MB-157), whereas Phenotype B comprised one pair derived from a non-effective cell line (Hs 578T).

This design illustrates how the framework retains methodological rigor while allowing flexibility, offering a practical template that can be readily adapted to investigate phenotype-specific responses across diverse biological and clinical contexts.

## Conclusion

In this study, we employed gene co-expression analysis to investigate the molecular mechanisms underlying the anti-tumor effects of the potent 4-(phenylsulfonyl)morpholine-based small molecule GL24. We implemented multiple data-driven strategies, including using *P* values as the sole significance threshold, excluding datasets with insufficient valid data points, and incorporating NA cases to explore the broadest range of potential gene co-expression. We also proposed potential patterns of differential gene pairs in the two effective cell lines, taking into account the high heterogeneity of TNBC, and excluded irrelevant gene associations using data from the non-effective cells. These designs yielded results that aligned with those from previous DEG-directed analyses while providing additional insights, such as highlighting pathways involved in metabolic alterations, proliferation, and migration or invasion. These findings enhance our understanding of TNBC pathways and offer valuable insights for the development of new drugs targeting TNBC.

## Supporting information

S1 Fig Comparison of phenotype-anchored gene pair counts and total identified gene numbers across four existing co-expression methods. The number of gene pairs that show differential co-expression in the GL24-effective cell lines and non-differential co-expression in the non-effective cell line was calculated for each differential scenario (DS1 to DS3) and for the four tools (WGCNA, GeneCoEx, CoExp, GeCoNet-Tool). Blue indicates scenarios involving differential co-expression (light to dark blue corresponds to DS1 to DS3), and yellow indicates scenarios involving non-differential co-expression.
(TIF)

S1 Data Interactive Cytoscape session files for network topologies. This file contains the complete interactive network models for the top 10 DS1 and DS2 clusters presented in Fig 3E and 3F.
(ZIP)

S1 Text Supplementary methods. Implementation and parameters of four co-expression methods: (1) WGCNA, (2) GeneCoEx, (3) CoExp, and (4) GeCoNet-Tool.
(DOCX)

S1 Table Classification of gene pairs across differential scenarios (DS1-DS3). Summary of all gene pairs assessed for co-expression status changes between DMSO and GL24 treatments in two GL24-effective TNBC cell lines (MDA-MB-231 and MDA-MB-157) and one non-effective cell line (Hs 578T). Each gene pair was categorized into one of three differential scenarios: DS1, gain of correlation (from non-significant/NA to significant); DS2, loss of correlation (from significant to non-significant/NA); DS3, retained significance with a significant change in correlation strength. Only gene pairs showing differential co-expression in both effective cell lines while remaining non-differential in the non-effective line were retained. Corresponds to Fig 2B-2D.
(XLSX)

S2 Table Network topology for DS1 and DS2 networks. Summary of the node (gene) degree and the cluster sizes of our undirected, unweighted co-expression networks constructed for DS1 and DS2. Corresponds to Fig 3A-3D.
(XLSX)

S3 Table Enriched GO terms and KEGG pathways for the top 10 clusters. For each of the top 10 clusters in DS1 and DS2, this table lists member genes, STRING-identified interacting proteins (PPI; confidence > 0.95), and significantly enriched Gene Ontology (Biological Process) and KEGG pathways (FDR < 0.05). Terms overlapping between DS1 and DS2, and those meeting FDR < 0.00001, are highlighted. Corresponds to Fig 4B-4E.
(XLSX)

S4 Table Enrichment statistics for the terms meeting FDR < 0.00001. This table provides the complete enrichment statistics for the top Gene Ontology (GO) biological processes and KEGG pathways presented in Fig 4D-4E.
(XLSX)
