# Supplementary figures and images for: Network-based exploration of 4-(phenylsulfonyl)morpholine molecules for metastatic triple-negative breast cancer suppression
Source: PLoS Comput Biol. 2026 Mar 31;22(3):e1014132. doi: 10.1371/journal.pcbi.1014132 (PMC13037966; doi:10.1371/journal.pcbi.1014132)

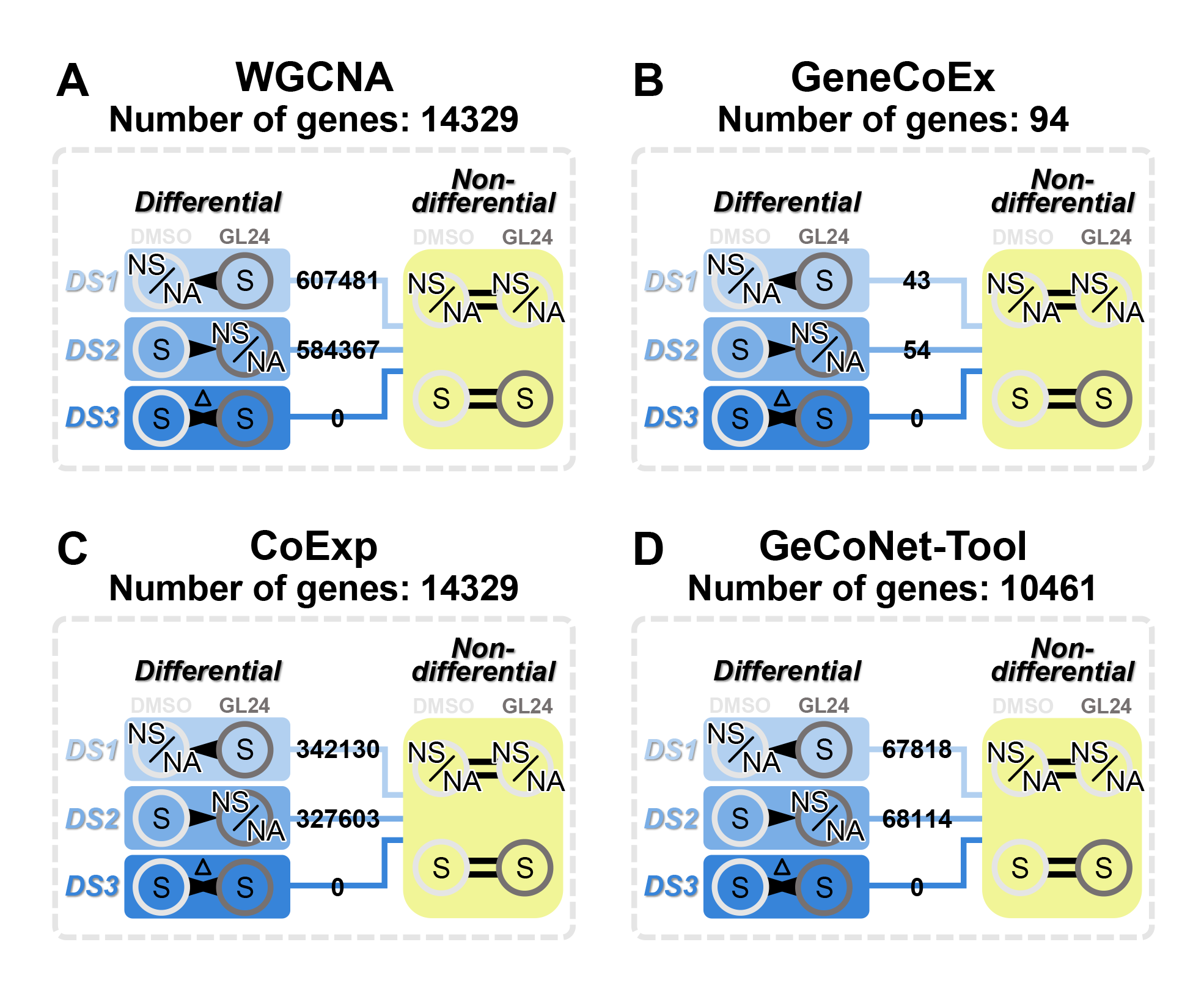

Supplement: S1 Fig — The number of gene pairs that show differential co-expression in the GL24-effective cell lines and non-differential co-expression in the non-effective cell line was calculated for each differential scenario (DS1 to DS3) and for the four tools (WGCNA, GeneCoEx, CoExp, GeCoNet-Tool). Blue indicates scenarios involving differential co-expression (light to dark blue corresponds to DS1 to DS3), and yellow indicates scenarios involving non-differential co-expression. (TIF) [file pcbi.1014132.s001.tif]
