# Supplementary material for: Network-based exploration of 4-(phenylsulfonyl)morpholine molecules for metastatic triple-negative breast cancer suppression
Source: PLoS Comput Biol. 2026 Mar 31;22(3):e1014132. doi: 10.1371/journal.pcbi.1014132 (PMC13037966; doi:10.1371/journal.pcbi.1014132)
Supplement: S1 Text — Implementation and parameters of four co-expression methods: (1) WGCNA, (2) GeneCoEx, (3) CoExp, and (4) GeCoNet-Tool. (DOCX) [file pcbi.1014132.s003.docx]

**S1 Text. Implementation and parameters of four co-expression methods**

**(1) WGCNA.** Weighted gene co-expression network analysis (WGCNA) was performed using the WGCNA R package (v1.73). Expression values were log_2_(x+1)-transformed and arranged as a samples-by-gene matrix prior to network construction. To select an appropriate soft-thresholding power, we applied the pickSoftThreshold() function to evaluate the scale-free topology fit index (R^2^) and mean connectivity across a range of candidate powers. Given the small sample size, higher thresholds (e.g., R^2^ ≥ 0.8) were not attainable, so we adopted the smallest power yielding R^2^ ≥ 0.45 for downstream analysis. Networks were constructed using the chosen soft-threshold, and the resulting adjacency matrix was transformed to a topological overlap matrix (TOM) for greater network robustness. Gene modules were identified by hierarchical clustering of the TOM.

**(2) GeneCoEx.** GeneCoEx analyses were conducted following the standard GeneCoEx pipeline. Expression values were log_10_(x+1)-transformed, and genes were variance-filtered, retaining the top 5,000 most variable genes. Pairwise gene-gene correlations were computed using Pearson correlation coefficients. *P* values were adjusted for multiple testing using the Benjamini-Hochberg procedure, and edges were retained at FDR < 0.05. All analyses were carried out in R (v4.5.1).

**(3) CoExp.** CoExp is primarily distributed as a web service and does not accept direct uploads of custom networks. Accordingly, we implemented the CoExp algorithm locally using the R package km2gcn (v0.1.0), following the procedures described in the original publication. km2gcn requires as input an expression matrix and initial module assignments derived from WGCNA; the WGCNA pipeline used here followed the procedure described above. Post-processing was performed with the function applykM2WGCNA(), which applies an iterative k-means reassignment: genes are reallocated to the module whose eigengene is closest (in correlation space); module eigengenes are recomputed and module membership is updated until convergence or until the maximum number of iterations is reached.

**(4) GeCoNet-Tool.** Analyses were performed using the GeCoNet-Tool software (v1.1). Enabled preprocessing options included log_2_-transformation, z-score normalization, and the removal of zero-only features. For analyses restricted to individual cell lines (*n* = 5 replicates), a bin size of 1 was utilized for the threshold-curve fitting step, with the cutoff value set to 0.05. While the threshold curve in GeCoNet-Tool is typically designed to identify cutoffs by fitting curves across bins based on the number of paired elements, the restricted sample size led the curve-fitting procedure to deterministically yield the minimum observed value. This value was then applied as the cutoff to the correlation matrix to derive the edge list.
